# The role of Exosomal miRNAs in cancer

**DOI:** 10.1186/s12967-021-03215-4

**Published:** 2022-01-03

**Authors:** Chuanyun Li, Tong Zhou, Jing Chen, Rong Li, Huan Chen, Shumin Luo, Dexi Chen, Cao Cai, Weihua Li

**Affiliations:** 1 Fengtai District, YouAn Hospital, Capital Medical University, NO. 8, Xitoutiao, Youanmen wai, Beijing, China; 2 Beijing Institute of Hepatology, Beijing, China; 3 Xiangya School of Medicine, Central South University, Changsha, China; 4 Chengde Medical University, Chengde, China

**Keywords:** Exosome, miRNAs, Cancer, Treatment resistance

## Abstract

Exosomal miRNAs have attracted much attention due to their critical role in regulating genes and the altered expression of miRNAs in virtually all cancers affecting humans (Sun et al. in Mol Cancer 17(1):14, 2018). Exosomal miRNAs modulate processes that interfere with cancer immunity and microenvironment, and are significantly involved in tumor growth, invasion, metastasis, angiogenesis and drug resistance. Fully investigating the detailed mechanism of miRNAs in the occurrence and development of various cancers could help not only in the treatment of cancers but also in the prevention of malignant diseases. The current review highlighted recently published advances regarding cancer-derived exosomes, e.g., sorting and delivery mechanisms for RNAs. Exosomal miRNAs that modulate cancer cell-to-cell communication, impacting tumor growth, angiogenesis, metastasis and multiple biological features, were discussed. Finally, the potential role of exosomal miRNAs as diagnostic and prognostic molecular markers was summarized, as well as their usefulness in detecting cancer resistance to therapeutic agents.

## Background

Reports assessing exosomes in relation to cancer biology have been on an exponential rise recently. Exosomes represent membrane-bound vesicles with diameters ranging between 30 and 150 nm, and are found in virtually all body fluids. Most cell types release these molecules into the extracellular space upon fusion with the cytoplasmic membrane [1]. Exosomal content involves unique miRNAs, mRNAs, DNA fragments, lipids and proteins, which genetically specify their cells of origin [2]. Exosomes are then taken up by other cells for subsequent modulation of recipient cells. Currently, miRNAs in exosomes are attracting increasing attention due to their contributions in recruiting and reprogramming important components of the tumor microenvironment. They are also considered to be essential in intercellular communication along with cell-to-cell contact-related signaling through the transfer of soluble molecules [3]. This represents an emerging concept, with a shallow understanding of exosomal miRNAs in general. The current review examined the biological properties of exosomes, discussed the proposed mechanisms of miRNA loading into exosomes, highlighted the mechanisms by which exosomal miRNAs affect tumor progression and outlined the potential use of exosomal miRNAs as biomarkers in multiple malignancies [4].

## Exosome formation and the biogenetic and sorting mechanisms of exosomal miRNAs

At present, exosomes have been found to be mainly generated by mechanisms dependent on or independent of endosomal sorting complex required for transport (ESCRT).ESCRT consists of different protein complexes and helper proteins, which participate in the formation of membrane-invagination and polyvesicle body (MVBs), and mediates the fusion of MVBs with cell membrane, resulting in the formation of exosomes. Meanwhile, IT has been found that MVBs can also be produced in a mechanism independent of ESCRT, enriching the research on exosome formation [5].

**Fig. 1:**
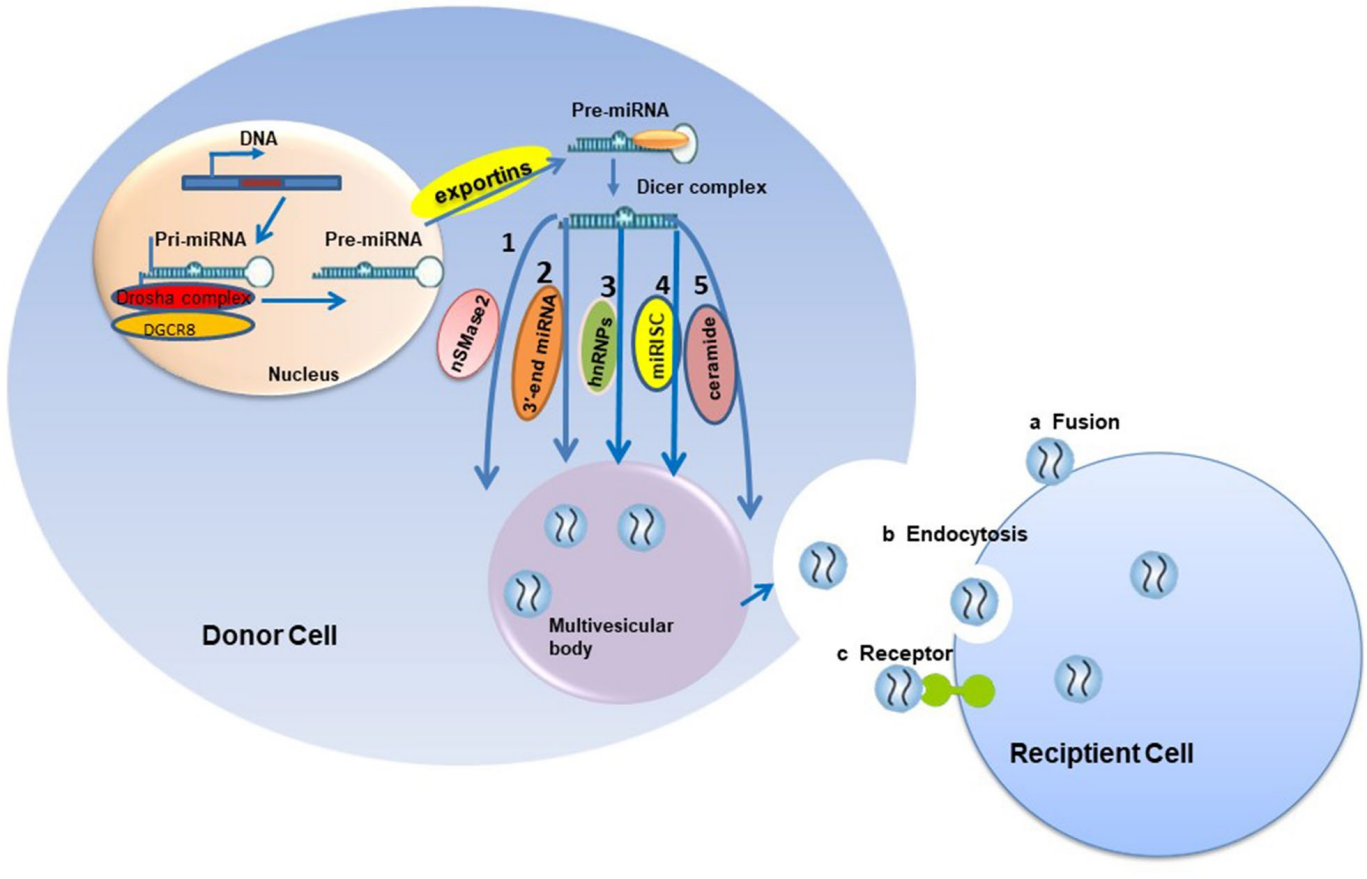
Sorting process for exosomal miRNAs. MiRNA genes undergo transcription into primary miRNAs (pri-miRNAs) by Pol-II, followed by processing by the DGCR8 and Drosha complex for the formation of precursor miRNAs (pre-miRNAs), which are exported into the cytosol by the exportin 5 complex. Then, pre-miRNAs undergo digestion by the Dicer complex into mature miRNAs, which are sorted into exosomes through five potential pathways: 1. nSMase2-dependent pathway; 2. 3′ miRNA sequence-dependent pathway; 3. miRNA motif and sumoylated hnRNPs-dependent pathway; 4. MiRNAISC-related pathway.5. Ceramide related pathway

The biogenetic process leading to exosomal miRNAs starts in the nuclear compartment, in which DNA sequences producing miRNAs undergo transcription by RNA polymerase for generating primary miRNAs (pri-miRNAs). The generated pri-miRNAs are initially included in longer molecules, and undergo processing in the nucleus to form 70–100 nt long hairpin RNAs [6]^.^ Exportin 5 assists in the transportation of hairpin pri-miRNAs to the cytoplasm, in which they are further processed by Dicer. Upon maturation, these double-stranded miRNAs are changed into single-stranded miRNAs, followed by miRNA sorting into exosomes. Based on the current research, it has been deduced that miRNA incorporation into exosomes is not a random event. Although the underlying mechanisms require clear understanding, the cells of origin use sorting processes that specifically guide intracellular miRNAs into exosomes [7]. There are about five potential channels performing miRNA sorting into exosomes, which are described below. (1) Neural sphingomyelinase 2 (nSMase2)-related pathway: nsMase2 was the first protein reported to be related to miRNA secretion into exosomes. Its downregulation decreased exosomal miRNA amounts, while its overexpression increased exosomal miRNA levels [8]. (2) The pathway associated with the 3′ ends of miRNAs. The 3′ ends of endogenous miRNAs containing uridines are mostly present in B cell- or urine-derived exosomes [9]. Therefore, the 3′ ends of miRNAs contain a powerful sorting signal. (3) The pathway associated with miRNA motifs and sumoylated heterogeneous nuclear ribonucleoproteins (hnRNPs). Sumoylated hnRNPA2B1 recognizes the GGAG motif at the 3′ end of miRNAs, causing the packaging of specific miRNAs into exosomes [10]. HnRNPA1 and hnRNPC are the other two hnRNP family members also binding to exosomal miRNAs, indicating their involvement in miRNA sorting. (4) MiRNA induced silencing complex (miRISC)-related pathway. Here, mature miRNAs and assembly proteins form the miRNAISC complex, which mainly contains miRNAs, miRNA-repressible mRNAs, and GW182 and AGO2 (Argonaute-binding proteins). The binding of mRNAs by these miRNAs occurs mostly within their 3′ untranslated regions (3′UTRs) [11]. The mRNA/miRNA complex next suppresses translation by preventing initiation or increasing mRNA degradation. Recently published findings have demonstrated a potential association of AGO2 with exosomal miRNA sorting. Data have indicated AGO2 knockout suppresses miRNAs such as miRNA-150, miRNA-142-3p and miRNA-451 in HEK293T-derived exosomes [10]. (5) Ceramide related pathway: As an exogenous ceramide supplement, ceramide C6(C6-cer) can dose-dependent inhibit the proliferation of human multiple myeloma (MM) cells and promote cell apoptosis Further studies showed that C6-cer treatment increased the levels of some tumor-suppressive mirnas (e.g. miR202,miR16,miR29b and miR15a) in exosomes, and the levels of these tumor-suppressive mirnas were also exosomes Changes also occurred in C6-cer treated MM cells, suggesting that the ceramide pathway plays an important role in miRNA incorporation into exosomes [12]. After miRNAs enter the exosomes, many reports have proposed mechanisms by which exosomes are secreted outside the cells, so this is not discussed in this review. The biogenetic and sorting mechanisms of exosomal miRNAs are presented in Fig. 1.

## Detection of exosome miRNA

Exosome miRNAs can be detected by a variety of methods, including quantitative reverse transcription polymerase chain reaction (qRT-PCR) 、fluorescence and ratio-based electrochemistry Local surface plasmon resonance (LSPR) quantitative methods for exosome miRNA without PCR, these methods enrich the detection technology of exosome miRNA to a certain extent, but there are still some problems such as poor RNA stability, complex and expensive technology and false positive signal [13]. In addition, precision medicine has made great progress in the medical field. The detection of circulating miRNA(C-miRNA) as part of precision medicine has been paid attention to C-miRNA as circulating targets (CTs) is readily available in a variety of body fluids and is released into body fluids in the form of miRNA protein complexes or EV-shuttle complexes, ensuring the high stability of C-miRNA during circulation and repeated freeze–thaw in long-term storage has been reported C-miRNA levels are stable under heating or extreme pH conditions, and thus can be used as a reliable CTs for liquid biopsy. Abnormal expression of C-miRNA can distinguish healthy patients from disease patients. In addition, C-miRNA helps to identify tumor origin and classify tumor types and subtypes, which are important for cancer diagnosis, treatment and prognosis Similarly, the detection of C-miRNA still has some problems, such as expensive equipment and complex operation, which still needs further exploration [14].

## The Functions of exosomal miRNAs in Cancer

### Exosomal miRNAs in tumor genesis and development

MiRNAs transported by exosomes can affect tumor growth and participate in various processes of tumorigenesis and tumor development [13]. In addition to Fas/FasL, transforming growth factor-β (TGF-β) and other factors, cancer cells secrete specific miRNAs in exosomes. These specific miRNAs, with immunosuppressive properties, can act on activated tumor specific T cells to promote the occurrence of tumors. For example, cervical cancer might be associated with miRNA-21 and miRNA-146a. These miRNAs can attenuate the targeting effect of cervical cancer-specific 293T cells as well as their killing effect on cancer cells, and can even induce 293T cell apoptosis. According to a study, the expression of miRNAs in the vaginal lavage fluid and exosomes of the HeLa cell line in vitro is overtly elevated compared with those of healthy controls, indirectly confirming the above conclusion [15].

In recent years, the emergence of the fluorescent exosome labeling technology has enabled breakthroughs in the relationships between exosome-derived miRNAs and tumor endothelial cells, angiogenesis, material information exchange, and tumor metastasis. For example, previous studies have confirmed that exosomes derived from breast cancer cells with high metastatic potential are rich in miRNA-105. When these exosomes are ingested by pulmonary microvascular endothelial cells, the expression of the tight junction protein ZO-I (Zonula occludens-I) in endothelial cells shows significant downregulation. This in turn leads to vascular permeability increase and helps tumor cells spread into the lungs, becoming a critical factor in tumorigenesis and cancer progression [16].

MiRNAs secreted by exosomes can mediate paracrine and endocrine pathways between different tissues to regulate gene expression and distal cell function. Exosome-derived miRNAs, which are paracrine agonists of toll-like receptor (TLR), are key regulators of tumorigenesis and cancer development. These are closely related to information exchange in the tumor immune system, with essential functions in tumorigenesis and cancer development. For instance, Fabbri and collaborators confirmed that exosomes produced by lung cancer cells contain miRNA-2 l and miRNA-129a, which in turn bind to TLR family members, including mouse TLR7 and human TLR8, as ligands on immune cells, triggering TLR-dependent inflammatory reactions and promoting lung cancer cell proliferation [17]. Some data suggest that miRNAs can regulate TLR signaling pathways through several steps, including regulating TLR mRNA expression and directly activating receptors to bind to TLR or TLR-specific signaling pathway components as well as TLR-induced transcription factors and functional cytokines At the same time, the recent discovery that exosomes contain miRNAs that can reach the TLR in the surrounding cell kernels provides new insights into physiological conditions and various regulatory mechanisms used in disease [18, 19].

In tumorigenic exosomes, there is a necessary cell-independent ripening of precursor miRNAs, which can still promote the malignant transformation of normal breast epithelial cells [20]. Therefore, whether exosomes can abandon tumor suppressor genes and retain cancer genes through unknown internal mechanisms remains unclear.

### Exosomal miRNAs in tumor invasion and metastasis

Exosomes obtained from tumor cells enhance the invasiveness of tumors via miRNA transportation [13]. For example, IL-4 activates the secretion of macrophages and transports the oncogenic miRNA-223 to target cells through the Mef2c-β-catenin (Myocyte-specific enhancer factor 2c) pathway. In this pathway, miRNA-223 affects β-catenin production in the nucleus of breast cancer cells by regulating its target gene Mef2c. This subsequently enhances breast cancer cell migration and invasion [21]. At the same time, exosomes act as carriers for the transportation of some miRNAs from the primary site to distant sites; activate tumor cells, endothelial cells and fibroblasts; and promote tumor metastasis [22]. For example, downregulation of tight junction proteins and barrier function damage in endothelial monolayers leads to the expression of cancer-secreted miRNA-105 in metastatic breast cancer, inducing vascular permeability and enhancing malignancy [23].

**Fig. 2:**
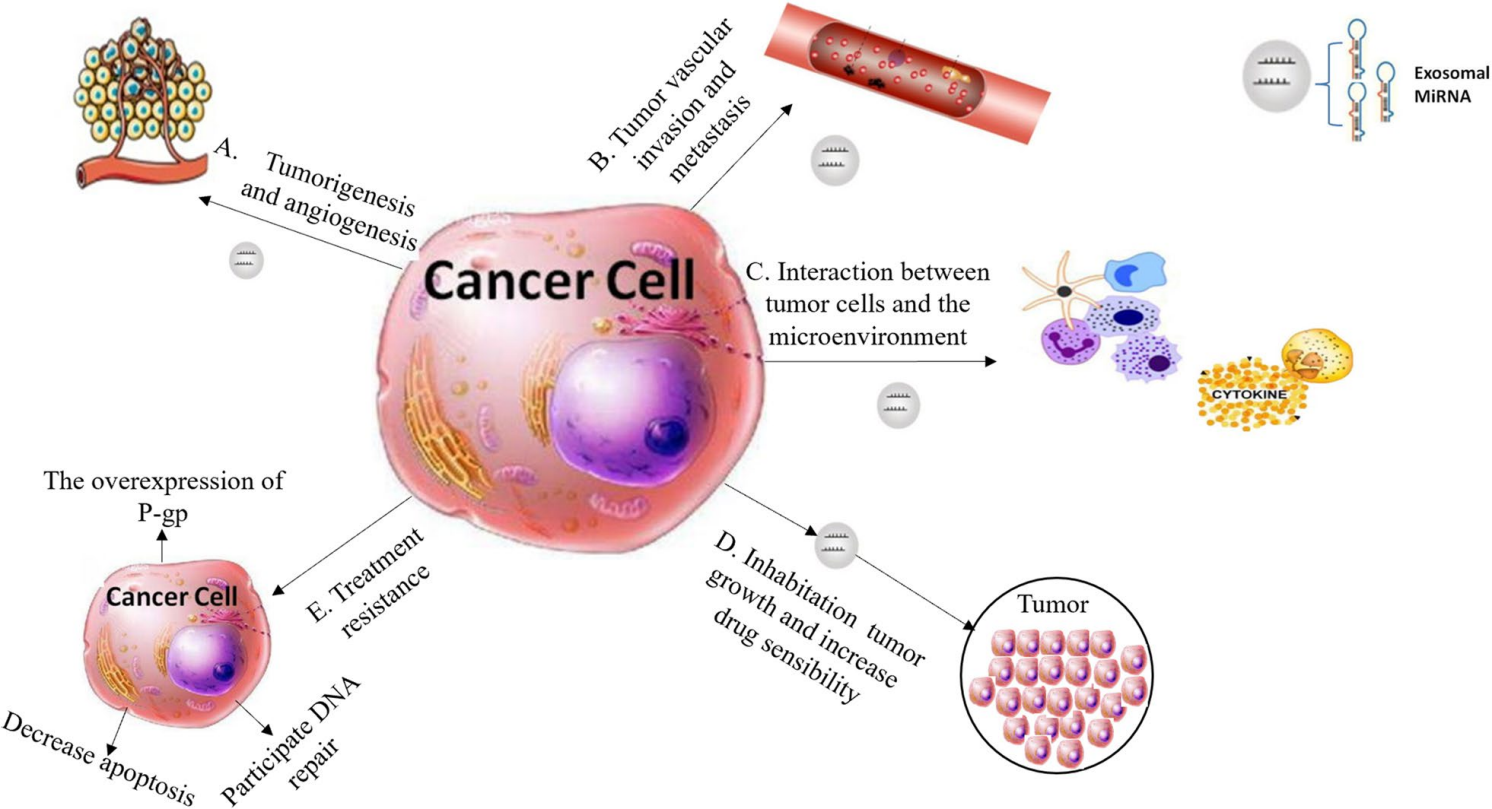
Functions of exosomal miRNAs in cancer. Exosomal miRNAs largely contribute to all carcinogenetic stages. The major mechanisms include the following. A. Tumorigenesis and cancer progression. B. Tumor vascular invasion and metastasis. C. Interactions of tumor cells with the surrounding microenvironment. D. Cancer treatment. E. Therapy resistance

In addition, exosomes could also be used as carriers to transport some miRNAs from the primary site to distant sites, activating tumor cells, endothelial cells and fibroblasts, and promoting tumor metastasis. Tanaka and co-workers detected miRNAs in serum exosomes from individuals with esophageal squamous cell carcinoma (ESCC) and benign esophageal lesions (without systemic inflammatory response) [24]. Their results showed overtly increased miRNA levels in ESCC cases compared with the control group. At the same time, cell experiments confirmed that serum exosomes from ESCC patients induce ESCC cell proliferation in vitro. The levels of miRNA-21 showed close associations with advanced esophageal cancer stage, lymph node metastasis, inflammatory reactions and so on. However, miRNA-21 could not be detected in the serum supernatant extracted from the exosomes. These results suggested some disease-related miRNAs are only specific to particular exosomes, and can achieve biological functions through the exosomal pathway. In addition to the indirect promotion of angiogenesis, tumor cell-derived exosomes also directly regulate target genes or signaling pathways in vascular endothelial cells by transferring miRNA-21, tissue factors and so on, thus increasing tumor malignancy. The functions of exosomal miRNAs in cancer are presented in Fig. 2.

### Exosomal miRNAs in the tumor microenvironment (TME)

The microenvironment around the tumor affects the malignant phenotype of the tumor. The TME includes tumor cells, cancer-associated fibroblasts (CAFs), and endothelial and immune cells, as well as non-cellular constituents, e.g., exosomes and cytokines, which also have essential functions [25]. Emerging evidence indicates cancer cells secrete higher amounts of exosomes compared with normal cells, even in the very early stage of carcinogenesis. Tumor-derived exosomes (TEXs) function as messengers controlling tumor cells within the TME, with critical roles from the initial tumor generation to the late stage of cancer [26]. TEXs were even found to have critical functions in the pre-cancer stage [27]. In addition, TEXs carrying biologically active miRNAs can reflect the unique features of different kinds of cancers, affecting tumor development [28].

### TLR-dependent inflammatory reactions and promoting lung cancer cell proliferation

Compared with the normal internal environment, the most significant feature of the TME is hypoxia. In the TME, under the condition of hypoxia, the infiltration of tumor-related macrophages is critical to tumor occurrence and development. It was reported by Wang et al. [29] that under the condition of hypoxia, macrophages are activated to acquire the M2 phenotype by pancreatic cancer cells through exosomal miR-301a-3p to activate PTEN/PI3Kγ signaling in macrophages. In addition, Chen et al. revealed exosomal miRNA-940 from epithelial ovarian cancer could promote M2 polarization under hypoxia. Other evidences also indicate under hypoxia miRNA-21-3p, miRNA-125b-5p, and miRNA-181d-5p could stimulate M2 polarization in macrophages [30]. M2 macrophage polarization would enhance cancer cell proliferation, invasion and migration. These findings reveal that under the condition of hypoxia, the profile of exosomal miRNAs is altered, which results in TME changes. In addition, many reports convincingly suggest miRNAs could function as oncogenes to structurally and biologically change the TME [31–34]. CAFs are an important part of the tumor microenvironment, and their interactions with cancer cells play an important role in the development and progression of cancer. Factors secreted by CAF include pro-inflammatory cytokines, such as IL-1β and IL-8, which are associated with pro-tumor effects. It has been found that tumor-derived exosome miRNA-1247-3p can induce the activation of tumor-associated fibroblasts and make them secrete cytokines such as IL-6 and IL-8 to promote lung metastasis of liver cancer [35]. Meanwhile, many research findings indicate exosome-associated miRNA-9 and miRNA-200 s can promote normal fibroblast (NF) transformation into CAFs and facilitate metastasis [36, 37]. Multiple vascular support is necessary for cancer progression and can provide nutrition and growth factors for tumors. Previous studies have shown that the most important function of exosomal miRNAs secreted by tumor cells is to affect vascular remodeling through IL-8-activated VCAM-1. Additionally, exosomes-associated miRNA-526b and miRNA-655 induce angiogenesis as well as lymphangiogenesis [38], while miRNA-340-5p and miRNA-561 induce an immunosuppressive TME [39, 40].

### Exosomal miRNAs in cancer treatment

Increasing evidence shows many small molecule substances can be effectively used to cure various cancers [41]. The key point is how to transport these substances to treat cancers at different sites. Many evidences indicate more than one trillion exosomes from other individuals have been injected to patients without matching inter-patient human leukocyte antigen (HLA), with no overt immune toxicity detected in these patients [41]. MicroRNAs are about 19 to 25-nucleotide long, single-stranded, non-coding RNAs encoded by endogenous genes, which can regulate gene expression to participate in vital cell processes [42]. Although there are currently no miRNA drugs approved for clinical treatment because of low stability, unexpected immune responses and poor cell membrane penetration, packing miRNAs in exosomes could overcome the latter limitations and would be a promising therapeutic tool in various malignancies [43]. Findings reported by Hamideh and colleagues confirmed miRNA (miRNA)-21-sponge packed in exosomes can effectively suppress the growth of brain tumors [44]. Another research revealed exosomal miRNA-123a could suppress tumor growth and improve survival in experimental mice [45, 46]. Furthermore, exosomes were used as carriers to transport miRNA-134 to breast cancer cells, to suppress tumor growth and enhance sensitivity to some drugs [47]. With the rapidly developing gene-editing technology, CRISPR-Cas9 combined with exosomes miRNAs would dramatically improve cancer treatment [48].

### Exosomal miRNAs in cancer resistance

In recent years many drugs have been approved for the clinical treatment of various cancers. However, the most significant reason for failed therapy is cancer cell resistance to therapeutics. The causes of drug resistance are mainly included in the following two points: (1) genetic and/or phenotypic alterations (intrinsic resistance); (2) interaction with the TME (extrinsic resistance) [49]. Exosomal miRNAs substantially contribute to both intrinsic and extrinsic resistance mechanisms (Hazlehurst and Dalton 2001; Meads et al. 2009). The detailed mechanism of therapy resistance can be summarized as anti-apoptotic signaling, enhanced DNA repair or ABC transporter delivery to drug-sensitive cells [49].

P-glycoprotein (P-gp), the most critical drug transporter known to date, is a product of the multidrug resistance protein 1 (MDR1) gene. Over 50% of cancers with the multiple drug resistance (MDR) phenotype express this protein [50]. P-gp’s half-life approximates 14-17 h; however, Levchenko et al. indicated exosomal P-gp lasting for about 4 months can confer tumor cells a longer acquired resistance phenotype [51]. Emerging evidence demonstrates P-gp-related exosomal miRNAs can lead to long-term P-gp expression in recipient cells [52]. For example, exosomal miRNA-451 and miRNA-27a are abundant in recipient cells, which could explain these long-term effects [53].

Exosomal miRNAs can regulate the cell cycle and cause anti-apoptotic pathways to confer resistance to therapy-sensitive tumor cells [52]. Via downregulation of the PTEN pathway, upregulated exosomal miRNA-222 can lead to cell cycle arrest in breast cancer cells, which can confer resistance to drug-sensitive tumors [23]. Another research demonstrated colorectal cancer cells can secrete exosomal miRNA-145/-34a that can decrease apoptosis to lead to 5-fluorouracil resistance of these cells [54].

According to Mutschelknaus et al. [55], exosomal miRNAs may participate in the progress of DNA repair, potentially leading to resistance in malignant cells. There are few reports examining the mechanism of exosomal miRNAs. Further studies should focus on this research area.

### Exosomal miRNAs as diagnostic and prognostic markers in cancer

The proportions of miRNAs are elevated in exosomes compared with their cells of origin [56]. As miRNAs in exosomes are shielded from RNase degradation because of lipid bilayer protection, they show higher stability in serum exosomes. Consequently, applying exosomal miRNAs as molecular markers for the diagnosis of diseases is an ongoing hot research topic. Exosomal miRNAs from malignant cells act as novel molecular markers to monitor cancer progression and treatment efficacy thanks to the following features: (1) exosomes secreted by cancer cells contain cancer-related proteins, RNAs and DNA fragments useful for cancer detection [57]; (2) the membrane structure of exosomes can enhance the stability of miRNAs, which in turn enhances their potential as biomarkers in diseases. Compared with miRNAs in cells and plasma, circulating exosome-derived miRNAs have a better anti-degradation ability [58]; (3) exosomes are broadly found in various body fluids (even the cerebrospinal fluid) and are easily accessible for clinical detection [59]; and (4) blood constituents are complex, which dilutes specific cancer-associated proteins, making it difficult to detect cancer proteins at an early stage or in case of low expression. More than 10^9^/ML circulating exosomes are found in humans. After purification, a large number of exosomes could be collected, from which cancer-associated exosomes are obtained. Consequently, exosomes can be detected in vivo with high sensitivity, enabling early diagnosis in cancer [60]. On the basis of the above characteristics, exosomal miRNAs have tremendous potential as molecular markers of cancer, which could help in diagnostic and prognostic possesses (Table 1).

**Table 1:** Exosomal miRNAs as prognostic molecular markers

Cancer	Exosomal miRNAs	Donor	Recipient	Type of biomarker	Refs.
Lung cancer	miR-21	Bronchial epithelial (HBE) cells	Normal HBE cells	Angiogenesis	[88]
	miR-23b-3p, miR-10b-5p and miR-21-5p	Plasmatic exosomes	Non-small cell lung cancer cells(NSCLC)	Progression, angiogenesis and metastasis	[61, 109]
	miR-210	Lung adenocarcinoma	Stromal cells	Angiogenesis	[110]
	miR-155/-146a	Immune cells	Immune cells	Inflammation	[111]
	miR-192	A549	Endothelial cells	Bone metastasis	[112]
	miR-494	Lung adenocarcinoma cells	Lymph nodes, lung cells	Pre-metastasis	[113]
Esophageal cancer(ESCC)	miR-30a	ESCC cells	/	Proliferation	[114]
Gastric cancer	miR-21	Macrophage	BGC-823	Metastasis	[115]
	miR-221	Mesenchymal stem cells	HGC-27	Metastasis	[116]
Colorectal cancer(CRC)	miR-19a	CRC cells	/	Metastasis	[117]
	miR-21, -192 and-221	HCT-15, SW480 and WiDr	HepG2 and A549	/	[118]
	miR-23b-3p	Blood plasma isolated from CRC patients	Colon cancer cells	Inhibitor	[109]
Liver cancer	miR-122	Huh7 cells	HepG2 cells	Inhibitor	[119]
	miR-223	Macrophages	Hepatocellular carcinoma cells (HuH7 and HepG2)	Inhibitor	[120]
Pancreatic cancer	miR-19b	PCa cells	Urine	Diagnosis	[121]
	miR-23b-3p	PANC-1 cells	PANC-1 cells	Proliferation and metastasis	[30, 109]
	miR-122-5p and miR-193b-3p	Plasma samples	Pancreatic cancer cells	Proliferation	[122]
	miR-141	PCa cells	Serum	Metastasis	[100]
	miR-141, miR-375	PCa cells	Serum	Metastasis	[98]
	miR-145	Urine	PCa cells	Suppressor	[98]
	miR-196a-5p	Urine	PCa cells	Metastasis	[123]
	miR-200c-3p	Urine	PCa cells	Suppressor	[98]
	miR-1246	Serum	PCa cells	Metastasis	[103]
	miR-1290, miR-375	PCa cells	Plasma	Survival prognosis	[124]
Bladder cancer	Exosome-derived miR- 29c	miR-29c	BIU-87 cells	Apoptosis	[125]
Prostate cancer	miR-34a	Docetaxel-resistant PC cells	Docetaxel-resistant	Drug resistance	[126]
	miR-125a	DIAPH3-silenced cells	macrophages	Proliferation	[126]
	miR-141	Bone metastatic PCa cells	Bone cells	Metastasis	[124]
	miR-375	Serum	PC cells	Metastasis	[124]
	miR-290,-378	PC cells	/	Prognosis	[127]
	miR-1290	Plasma	/	Prognosis	[124]
Ovarian cancer	miR-21-5p	CP70	A2780	Drug resistance	[128]
	miR-127-3p	/	OVCAR-3 and Caov-3 cells	Proliferation	[129]
	miR-200a/b/c/141	SKOV-3 and OVCAR-3	Ovarian cancer cells (OC)	Proliferation	[103]
	miR-339-5p	OC ES-2 cells	Endothelial cells	Proliferation and metastasis	[129]
	miR-409-3p	OC ES-2 cells	Endothelial cells	Proliferation and metastasis	[129]
Breast cancer	miR-16	EGCG-treated 4 T1 cells	Macrophages	Metastasis	[130]
	miR-21	Bone marrow-derived MSCs	Breast cancer cells	Proliferation	[131]
	miR-23b	Bone marrow mesenchymal stem cells	Breast cancer cells	Dormancy	[132]
	miR-105	MDA-MB-231	Endothelial cells	Metastasis	[133]
	miR-10b	MDA-MB-231	HMLE (MCF-7)	Metastasis	[134]
	miR-122	Breast cancer patients/ MCF10A	Recipient pre-metastatic niche cells	Metastasis	[135]
	miR-134	Hs578T and Hs578Ts(i)8	Breast cancer cells	Drug resistance	[30]
	miR-200	Metastatic breast cells	Non-metastatic breast cells	Metastasis	[136]
	miR-221/ -222	MCF-7 (Tamoxifen resistant)	MCF-7 (Tamoxifen-sensitive)	Drug resistance	[137]
	miR-223-3p	IL-4-activated macrophages	MDA-MB-231	Metastasis	[138]
	MiR-373	breast cancer cells	/	Malignant prediction	[127]
	miR-503	Endothelial cells	Breast cancer cells	Metastasis	[139]
Neuroblastoma	miR-21	NBL cells	Human monocytes	Drug resistance	[124]
Hematological malignancies	miR-21	CLL cells	MSCs and endothelial cells	Metastasis	[140]
	miR-92a	K562 cells	Umbilical vein endothelial cells	Metastasis	[18]
	miR-126	LAMA84	Endothelial cells	Metastasis	[141]
	miR-135b	Multiple myeloma cells	endothelial cells	Metastasis, angiogenesis	[142]
	miR-202-3p	Chronic lymphocytic leukemia (MEC1)	Human stromal cells	Proliferation	[143]
	miR-210	K562 under hypoxic conditions	Umbilical vein endothelial cells	Metastasis	[144]
Melanoma	miR-31, -185, and -34b	A375 and SK-MEL-28	Normal melanocytes	Metastasis	[145]
Melanoma	miR-125b-5p	PLX4032-resistant melanoma cell line	Primary melanoma cell lines	Metastasis	[146]
	miR-222	Metastatic melanoma cell lines	Primary melanoma cell lines	Metastasis	[147]
Merkel Cell Carcinoma (MCC)	miR-30a, miR-34, miR-142-3p, miR-1539	MCV-positive or -negative tumors	/	MCPyV infection	[148]

## Expression of exosomal miRNAs in cancers

### Respiratory system cancer

Previous studies have reported the importance of exosomes in the pathogenetic mechanisms of lung cancer, from initiation to metastasis [61]. Exosomes contribute to multiple functions and intrinsic pathways in malignant cells, and are considered valuable molecular markers [62]. Recent evidence suggests exosomal miRNAs could be utilized as diagnostic markers in lung cancer [58, 63, 64].

MiRNA-361-3p and miRNA-625 found in the blood assisted in differentiating malignant lung cancer from benign lung lesions [65]. In addition, upregulation of miRNA-21 in the blood was shown to be a reliable biomarker for the early diagnosis of squamous cell lung cancer. MiRNA-122-5p is markedly elevated in exosomes from lung cancer cases compared with the bronchoalveolar lavage fluid [66]. The levels of serum miRNA-200b-5p, miRNA-378, miRNA-502-5p, miRNA-629, miRNA-17 and miRNA-100 are remarkably higher in individuals with lung adenocarcinoma compared with pulmonary granuloma cases as well as healthy smokers [67]. High miRNA-21 and miRNA-155 amounts in lung cancer exosomes have been considered potential biomarkers for diagnosing lung cancer [68, 69]. The levels of miRNAs, including miRNA-205, miRNA-19a, miRNA-19b, miRNA-30b and miRNA-20a, were shown to be significantly decreased after operation [70]. Extraction of exocrine miRNAs from peripheral blood, BALF and other bodily fluids can be used to detect miRNAs, and exhaled gas concentrates can also contain biomarkers that may help diagnose lung diseases [71]. In proteomics-based mass spectrometry analysis, urinary exosomes were remarkably more efficient in expressing leucine-rich alpha-2-glycoprotein 1 (LRG1) in non-small cell lung cancer (NSCLC) cases compared with normal control individuals. Munagala and colleagues [72] studied serum exosomal miRNAs for diagnosing recurrent lung cancer. They observed expression changes for 77 miRNAs in in vitro cell culture and animal models. Of these, 47 were upregulated and 30 were downregulated. MiRNA-21 and miRNA-155 showed significant upregulation in recurrent tumors in comparison with primary tumors. These data suggested serum exogenous miRNAs might be used for non-invasive diagnosis of primary and recurrent lung cancers. In recent years, peripheral blood exosomes were shown to have 30 specific molecular markers. Thus, exosomes and associated molecules may provide a theoretical basis for determining biomarkers for diagnosing lung cancer at an early stage.

### Digestive system cancer

Gastrointestinal cancer represents one of the commonest malignancies. Nearly 300,000 people die of gastrointestinal cancer every year in the world. The severity of digestive tract cancer lies not only in its high morbidity and mortality rates, but also in the fact that 85% of digestive tract cancer cases are diagnosed in the middle and late stages. The probability of cure is often very low in this cancer type. Therefore, early diagnosis is the most effective way to reduce the mortality rate of patients with digestive tract cancer. According to recent reports, exosomes could provide means for early diagnosis in gastrointestinal cancer.

#### Liver cancer and cholangiocarcinoma

A recent report demonstrated hepatocellular carcinoma (HCC) cases have significantly increased levels of exosomal miRNA-665 compared with healthy individuals. In the latter patients, the levels of exosomal miRNA-665 showed relationships with tumor size and clinical stage. This suggested that exosomal miRNA-665 may be a suitable biomarker for HCC diagnosis and prognosis [73]. Sugimachi [74] compared serum exosome-derived miRNA levels between patients with HCC and normal people, and found serum miRNA-718 amounts were significantly decreased in HCC cases. Another study showed miRNA-638 suppression has negative associations with tumor size, vascular invasion and tumor stage, predicting low patient survival in HCC [73]. In another research, serum exosomal miRNA-122, miRNA-148a and miRNA-1246 amounts were starkly elevated in HCC in comparison with liver cirrhosis (LC) cases and healthy control (NC) groups [73]. Another work revealed that miRNA-101, miRNA-106b, miRNA-122 and miRNA-195 amounts in serum exosomes are significantly reduced in HCC cases compared with chronic hepatitis B (CHB) patients, while miRNA-18a, miRNA-222, miRNA-221 and miRNA-224 were markedly elevated in HCC cases compared with CHB or LC patients. Furthermore, serum-circulating miRNAs showed no significant differences between CHB and HCC cases. The above data suggested serum exosomal miRNAs could better distinguish HCC from CHB or LC in comparison with serum miRNAs [75, 76]. A report showed serum exosomal miRNA-21 levels not only are higher in HCC cases compared with CHB or healthy control individuals, but are also closely correlated with cirrhosis and advanced tumor stage. Although circulating miRNA-21 showed a similar trend, it was less sensitive in detecting tumors than serum exosomal miRNA-21. These findings demonstrated serum exosomal miRNA-21 could act as a new index with higher sensitivity compared with whole serum miRNA-21 in HCC detection [76–79].

#### Gastric cancer

Gastric cancer (GC) is one of the deadliest cancers, with high incidence worldwide. GC ranks fourth and second among cancers in terms of global incidence and mortality, respectively [80]. As carriers, exosomes have critical functions in interactions among malignant cells, vascular endothelial cells and macrophages. GC cell-derived exosomes activate NF-κB signaling in macrophages to induce tumor progression [81]. It was shown metastatic GC AZ-P7a cells release let-7 miRNA through exosomes for maintaining oncogenesis [82]. Elevated amounts of let-7 miRNA family members in AZ-P7a cell-derived exosomes might reflect GC metastasis. CD97 induces the proliferative and invasive potential of GC cells in vitro via exosome-mediated MAPK signaling, likely involving exosomal miRNAs [83].

It is imperative to determine the potential role of exosomal miRNAs in the peritoneal fluid in predicting GC dissemination in the peritoneum. Exosomal miRNA amounts were assessed by Agilent Human miRNA microarrays and quantitative reverse transcription polymerase chain reaction (qRT-PCR) in 24 peritoneal lavage fluid (PLF) specimens, 6 gastric malignant ascites (MA) samples and culture supernatants (CM). The data identified 5 miRNAs (miRNA-1225-5p, miRNA-320c, miRNA-1202, miRNA-1207-5p, and miRNA-4270) with elevated amounts in MA specimens, PLF samples from serosa-invasive GC cases, and CM of highly metastatic GC cells. MiRNA-21 and miRNA-1225-5p also showed an association with serosal invasion in GC. These results suggested that miRNA-21 and miRNA-1225-5p could represent molecular markers of peritoneal recurrence following curative GC resection. Thus, a new strategy for detecting peritoneal dissemination of GC in the early stage has been put forward [84].

#### Colorectal cancer

Microarray analysis was performed in 88 primary colorectal cancer cases and 11 control subjects with serum samples rich in exogenous miRNAs [85]. Differences in microarray analysis between the experimental and control groups were compared with the data of 29 patients after tumor resection. Meanwhile, the miRNAs retained as molecular markers were compared to CA-199 and carcinoembryonic antigen (CEA) for sensitivity. The results showed that the amounts of 7 miRNAs, i.e., let-7a, miRNA-1229, miRNA-1246, miRNA-150, miRNA-21, miRNA-223 and miRNA-23a, were markedly elevated in patients with primary colorectal cancer compared with healthy controls, but starkly reduced upon tumor resection. In stage I colorectal cancer patients, miRNA-23a and miRNA-1246 had higher sensitivities compared with CA-199 and CEA, i.e., 95.5% and 92%, respectively. The exosomal amounts of the miRNA-17-92a cluster in serum showed a correlation with colorectal cancer recurrence. Serum exosomal miRNA-19a amounts were remarkably elevated in colorectal cancer cases in comparison with healthy controls. In addition, colorectal cancer cases with elevated exosomal miRNA-19a amounts showed poorer prognosis in comparison with the low expression group. It has also been found that tumor-derived exosome Mir-934 induces macrophage M2 polarization and promotes liver metastasis of colorectal cancer [86].

In a research assessing tumor markers [87], let-7a, miRNA-1229, miRNA-1246, miRNA-150, miRNA-21, miRNA-223 and miRNA-23a in exosomes showed close associations with the occurrence of colon cancer. A comparison of 27 recurrence cases with 57 patients without recurrence identified about 145 differentially miRNAs. MiRNA-4772-3p had significant downregulation, which showed a significant correlation with elevated risk of disease recurrence and poor prognosis. Low amounts of serum exosomal miRNA-4772-3p could predict tumor recurrence in stage-II and III colon cancer [88].

#### Pancreatic cancer

Studying gastrointestinal cancer [89] showed that a variety of miRNAs in serum exosomes, and found that the expression of miRNA-1246, miRNA-4644, miRNA-3976 and miRNA-4306 was increased significantly in pancreatic cancer patients, while the expression of these miRNAs in the control group remained very low. CA19-9 is still regarded as the serum biomarker with highest reliability in diagnosing pancreatic cancer, but can be combined with other diagnostic tools, including serum miRNAs in pancreatic cancer patients to differentiate between benign and malignant tumors or inflammation.

### Nervous system Cancer

According to a recent study, serum exosomal miRNAs in schizophrenia and bipolar disorder cases showed significant variations from those of matched controls [90, 91]. In addition, schizophrenic individuals had high exosomal amounts of miRNA-497; similarly, increased exosomal amounts of miRNA-29c were detected in bipolar disorder cases. In studies assessing biomarkers showing correlations with Alzheimer’s disease (AD) diagnosis, a 7-miRNA AD-associated signature was derived using machine learning to predict AD status with 83 to 89% accuracy [92]. Studies identifying neurodegeneration-associated exosomal miRNAs in the cerebrospinal fluid might analyze the usefulness of exosomes as potential biomarker carriers in neurodegenerative pathologies. MiRNA-193b overexpression could suppress amyloid precursor protein (APP) expression, showing that miRNA-193b might contribute to neurodegeneration, with a potential to act as an important AD biomarker [93]. Furthermore, miRNA-193b was detected in hippocampal specimens from AD mice. Intriguingly, in a study examining Parkinson’s disease (PD), profiling of exosomal miRNAs was effective in differentiating PD and AD [94]. Exosomal miRNA biomarkers may assist in predicting future cognitive declination in asymptomatic people as well as disease progression in early dementia cases.

### Genitourinary system cancers

#### Renal cell carcinoma

Research on miRNAs in renal cell carcinoma mainly describes their importance in differentiating tumor tissues from the renal parenchyma, histological classification and renal cell carcinoma prognosis. A study discussed the detection of urinary miRNAs in patients with renal cell carcinoma [95]. As a suppressor gene in renal cell carcinoma, miRNA-15a promotes apoptosis and inhibits cell proliferation by closely binding to α-isoprotease C (PKCα). PKCα directly inhibits the release from the nucleus of pri-microNA-15a, which is the primary transcript of miRNA-15a, reduces PKCα, and increases the levels of miRNA-15a. In biopsy and urine samples, upregulation of miRNA-15a might also act as an important index for differentiating clear cell renal cell carcinoma from noncancerous kidney tumors.

#### Prostate cancer

A previous study reported exosomal miRNA-1246 is promising in detecting prostate cancer, and has a potential in predicting disease aggressiveness [96]. Another study showed plasma exosomal miRNA-1290 and miR-320a-3p and miR-155-5p act as potent markers for predicting prognosis in castration-resistant prostate cancer (CRPC) [97]. However, prospective validation is further needed to develop these candidate miRNAs. In addition, Cox regression analysis revealed associations of miRNA-1290, miRNA-1246 and miRNA-375 with overall survival (OS). High miRNA-1290 and miRNA-375 amounts showed significant associations with reduced OS during follow-up. Incorporating miRNA-1290/miRNA-375 into potential clinical prognostic factor-based models in Castration-Resistant Prostate Cancer stage significantly improved the predictive performance.

Bryant and colleagues [98] carried out RT-PCR to examine various miRNAs in plasma exosomes from 78 prostate cancer cases and 28 healthy controls, and showed that 11 miRNAs had significant increases in prostate cancer. MiRNA-141 and miRNA-375 might be used as diagnostic markers in prostate cancer [99]. Li and collaborators [100] assessed the feasibility of exogenous miRNAs in prostate cancer diagnosis. They separated and extracted exosomal miRNAs from serum specimens obtained from prostate cancer cases, individuals with benign prostatic hyperplasia and healthy people to analyze the levels of miRNA-141. As a result, miRNA-141 amounts in the prostate cancer group were significantly higher than those of the benign prostatic hyperplasia and control groups, and miRNA-141 amounts were markedly elevated in metastatic prostate cancer cases compared with non-metastatic prostate cancer cases. Therefore, exosomal miRNA-141 might be considered a good diagnostic and differential tool in urinary system tumors.

#### Ovarian cancer

Taylor's team was among the first to show that circulating exosomal miRNAs could be utilized to diagnose ovarian cancer [101]. They found significant differences in the miRNA profiles of blood exosomes between ovarian cancer cases and healthy individuals, indicating that exosomes may assist in diagnosing ovarian cancer. Moreover, it was found that miRNAs isolated from tumor-derived exosomes in serum show a good correlation with those extracted from ovarian cancer tissues. In addition, the miRNAs profiles of patient and normal control groups were analyzed, and the results showed significant differences. There were 8 miRNAs (miRNA-21, miRNA-141, miRNA-200a, miRNA-200b, miRNA-200c, miRNA-203, miRNA-205 and miRNA-214) considered to be useful for the early diagnosis of ovarian cancer. No significant differences in serum amounts were detected between early and advanced ovarian cancer cases, but benign and malignant tumors showed overt differences. These results indicated exosomal miRNAs could act as a useful tool for diagnosing ovarian cancer early. Serum miRNA-1290 showed significant elevation in individuals with high-grade serous ovarian cancer, and could distinguish these patients from individuals with cancers of different histological types. Thus, miRNA-1290 is regarded as a novel biomarker for HGSOC detection [102].

Meng and co-workers [103] measured the levels of miRNAs in serum exosomes from epithelial ovarian cancer cases. As a result, total exosomal miRNA expression was markedly elevated in ovarian cancer cases compared with healthy women, and miRNA-373, miRNA-200a, miRNA-200b and miRNA-200c were highly regulated. Therefore, miRNA-200a, miRNA-200b and miRNA-200c might be used to differentiate benign from malignant tumors, and the elevated serum levels of miRNA-200b and miRNA-200c showed close associations with CA125 and shorter survival. Generally speaking, the amounts of exogenous miRNAs are closely related to ovarian cancer development.

A study [104] demonstrated serum exosomal miRNA-375 and miRNA-1307 are markedly upregulated in ovarian cancer cases in comparison with the ovarian benign tumor and healthy groups. MiRNA-375 and miR-1307 are associated with lymph node metastasis and tumor staging in ovarian cancer, respectively. The upregulated miRNAs had independent diagnostic potential and increased the diagnostic accuracy of routine molecular markers such as CA-125 and HE4.

In high risk cervical cancer patients, well-run screening programs resulted in sharply decreased cervical cancer incidence and mortality [105]. Multiple cancers actively release miRNAs carried by exosomes. This review assessed the associations of miRNAs with cervical cancer-derived exosomes, which are abundant in the cervico-vaginal lavage in which exosomal miRNAs could be detected by electron microscopy, RT-qPCR and miRNA target reporter assays. MiRNA-21 and miRNA-146a, upregulated in cervical cancer cases, showed an association with elevated amounts of cervical cancer-derived exosomes. These findings suggested abnormally elevated miRNA-21 and miRNA-146a amounts in cervical cancer-derived exosomes, with the above two miRNAs playing functional roles in 293T cells.

### Other cancers

Roccaro and colleagues [106] analyzed the miRNA compositions of exosomes from bone marrow mesenchymal stem cells in healthy individuals and multiple myeloma cases by the miRNA chip assay. As a result, miRNA-15a amounts in the multiple myeloma group were significantly downregulated, which requires further exploration. Suo and colleagues found close correlations with the characteristics of multiple myeloma.

The levels of miRNA-1246 and miRNA-21 in plasma exosomes of breast cancer cases were significantly increased in comparison with those of healthy controls. Receiver operating characteristic (ROC) curve analysis revealed combined plasma exosomal miRNA-1246 and miRNA-21 levels better reflected breast cancer than each individual level alone [23, 107]. Another study showed that exosomal miRNA-105 amounts are elevated in metastatic breast cancer cases compared with non-metastatic cases and healthy controls. Similarly, elevated miRNA-222 amounts were detected in luminal A versus luminal B tumor subtypes as well as in basal-like cells. Exosomal miRNA-222 amounts showed correlations with clinical and pathological parameters. During treatment, the levels of exosomal miRNA-21 had a direct correlation with tumor size and an inverse correlation with Ki67 amounts. In addition, high exosomal miRNA-21, miRNA-155 and miRNA-222 amounts were closely associated with the presence of circulating tumor cells.

## Future perspectives

Diagnosing cancer at an early stage could reduce disease-related deaths and extend the lifespan. Exosomes have become a hot research topic as a biomarker for diagnosing multiple diseases. Previous researches have revealed that miRNAs enclosed in exosomes could escape immune attacks and cross the blood–brain barrier. In addition, exosomes prevents the clearance and/or damage of miRNAs by complement fixation and macrophages because of their double-layered membrane and size in the nanometer range, prolonging their circulatory half-life and improving clinical diagnosis. Mounting evidence indicates exosomal miRNAs are circulating diagnostic markers for different types of cancer [108]. The unique features of miRNAs in exosomes could be modified to increase their diagnostic efficacy for use as ideal tools for non-invasive screening and targeting of cancer cells in clinic. Several reviews discussing serum exosomal miRNAs in multiple malignancies are available. However, utilizing exosomes and their cargo as a diagnostic and monitoring tool for diseases remains challenging, requiring further investigation. Meanwhile, accurate purification, identification and high-throughput use of extracellular vesicles in clinic face enormous challenges. Further developing tumor exosomal miRNAs and improving microfluidics for exosome detection could improve their use for diagnosing cancer.

## Data Availability

Not applicable.

## Abbreviations

AD: Alzheimer’s disease
APP: Amyloid precursor protein
CAF: Cancer-associated fibroblast
CEA: Carcinoembryonic antigen
CHB: Chronic hepatitis B
CM: Culture supernatants
CRISPR: Clustered regularly interspaced short palindromic repeats
CRPC: Castration-Resistant Prostate Cancer
ESCC: Esophageal squamous cell carcinoma
GC: Gastric cancer
HCC: Hepatocellular carcinoma
HLA: Human leukocyte antigen
hnRNP: Heterogeneous nuclear ribonucleoprotein
LC: Liver cirrhosis
LRG1: Leucine-rich alpha-2-glycoprotein
MA: Malignant ascites
MAPK: Mitogen-activated protein kinase
MDR: Multidrug resistance
Mef2c: Myocyte-specific enhancer factor 2c
miRISC: MiRNA induced silencing complex
NC: Normal control
NF: Normal fibroblast
NSCLC: Non-small cell lung cancer
nSMase2: Neural sphingomyelinase 2
PD: Parkinson’s disease
PI3K: Phosphatidylinositol 3 kinase
PKCα: α-Isoprotease C
PLF: Peritoneal lavage fluid
pri-miRNAs: Primary miRNAs
PTEN: Phosphatase and tensin homolog deleted on chromosome ten
TEXs: Tumor-derived exosomes
TGF-β: Transforming growth factor-β
TLR: Toll-like receptor
TME: Tumor microenvironment
ZO-I: Zonula occludens-I
